# Genome-Wide Analysis of the GRAS Gene Family and Functional Identification of *GmGRAS37* in Drought and Salt Tolerance

**DOI:** 10.3389/fpls.2020.604690

**Published:** 2020-12-23

**Authors:** Ting-Ting Wang, Tai-Fei Yu, Jin-Dong Fu, Hong-Gang Su, Jun Chen, Yong-Bin Zhou, Ming Chen, Jun Guo, You-Zhi Ma, Wen-Liang Wei, Zhao-Shi Xu

**Affiliations:** ^1^College of Agriculture, Yangtze University, Jingzhou, China; ^2^Hubei Collaborative Innovation Center for Grain Industry, Yangtze University, Jingzhou, China; ^3^Engineering Research Center of Ecology and Agricultural Use of Wetland, Ministry of Education, Yangtze University, Jingzhou, China; ^4^Institute of Crop Science, Chinese Academy of Agricultural Sciences (CAAS)/National Key Facility for Crop Gene Resources and Genetic Improvement, Key Laboratory of Biology and Genetic Improvement of Triticeae Crops, Ministry of Agriculture, Beijing, China; ^5^Institute of Urban Agriculture, Chinese Academy of Agricultural Sciences, Chengdu, China; ^6^State Key Laboratory of Crop Stress Biology for Arid Areas, College of Plant Protection, Northwest A&F University, Yangling, China

**Keywords:** GRAS protein, genome-wide analysis, abiotic stress, hairy root assay, soybean

## Abstract

*GRAS* genes, which form a plant-specific transcription factor family, play an important role in plant growth and development and stress responses. However, the functions of *GRAS* genes in soybean (*Glycine max*) remain largely unknown. Here, 117 *GRAS* genes distributed on 20 chromosomes were identified in the soybean genome and were classified into 11 subfamilies. Of the soybean *GRAS* genes, 80.34% did not have intron insertions, and 54 pairs of genes accounted for 88.52% of duplication events (61 pairs). RNA-seq analysis demonstrated that most *GmGRASs* were expressed in 14 different soybean tissues examined and responded to multiple abiotic stresses. Results from quantitative real-time PCR analysis of six selected *GmGRASs* suggested that *GmGRAS37* was significantly upregulated under drought and salt stress conditions and abscisic acid and brassinosteroid treatment; therefore, this gene was selected for further study. Subcellular localization analysis revealed that the GmGRAS37 protein was located in the plasma membrane, nucleus, and cytosol. Soybean hairy roots overexpressing *GmGRAS37* had improved resistance to drought and salt stresses. In addition, these roots showed increased transcript levels of several drought‐ and salt-related genes. The results of this study provide the basis for comprehensive analysis of *GRAS* genes and insight into the abiotic stress response mechanism in soybean.

## Introduction

Abiotic stresses, such as drought, heat, cold, and salt, seriously affect plant growth and development. Some transcription factors respond to adverse conditions by binding to specific DNA sequences in target promoters to regulate the transcription level of the target genes (Riano-Pachon et al., 2007; Liu et al., 2018; Song et al., 2019). Studying plant transcription factors can help us understand the regulatory network underlying various biological processes. The GRAS group, which is named from the three initially determined members, *GIBBERELLIN-ACID INSENSITIVE* (*GAI*), *REPRESSOR of GA1* (*RGA*), and *SCARECROW* (*SCR*; Di Laurenzio et al., 1996; Peng et al., 1997; Silverstone et al., 1998).

The C-terminal region of GRAS proteins is highly conserved and is commonly referred to as the GRAS domain (Pysh et al., 1999). GRAS transcription factors usually have only one GRAS domain, but a few GRAS proteins have two GRAS domains or one GRAS domain and another functional domain. The GRAS domain can be divided into five units: Leucine heptad repeat I (LHRI), Leucine heptad repeat II (LHRII), VHIID, PFYRE, and SAW (Bolle, 2004). However, the N-terminal amino acid sequences of GRAS proteins are highly variable and may determine the specificity of these regulatory proteins (Tian et al., 2004).

GRAS family members have been identified in multiple plants, and the family classification is slightly different between species (Liu and Widmer, 2014). For example, 47 *GRAS* genes of Tartary buckwheat were divided into 10 subfamilies: DELLA, DLT, HAM, PAT1, LAS, LISCL, SCR, SCL3, SHR, and SCL4/7 (Liu et al., 2019). However, 150 *GRAS* genes in upland cotton have been classified into 14 subfamilies, namely DLT, DELLA, HAM, PAT1, LAS, SHR, LISCL, SCR, SCL3, SCL4/7, Os19, Os4, OS43, and G_GRAS (Zhang et al., 2018). In tomato, 53 *GRAS* genes were divided into 13 subfamilies: PAT1, SHR, SCL9, Os4, GRAS37, Pt20, DELLA, SCR, Os19, SCL28, SCL4/7, LAS, and HAM (Huang et al., 2015). These studies indicate that this gene family diversified substantially in different species plants. The classification of members of the *GRAS* gene family reflects its evolutionary history.

Different subfamilies may have different functions during plant growth. According to previous reports, GRAS family members are essential for physiological processes such as gibberellic acid signal transduction, stem cell maintenance, axillary meristem initiation, light signaling, phytochrome signaling, male gametogenesis, and detoxification (Dill et al., 2001; Stuurman et al., 2002; Greb et al., 2003; Li et al., 2003; Lee et al., 2008). For example, the DELLA subfamily participates in gibberellin signaling in *Arabidopsis* (Peng et al., 1997); the AtLAS subfamily in axillary meristem development of tomato (Schumacher et al., 1999); the HAM subfamily in shoot meristem sustention in petunia (Stuurman et al., 2002); the AtPAT1 subfamily in phytochrome A signaling in *Arabidopsis* (Bolle et al., 2000); the LISCL subfamily in transcriptional regulation during microsporogenesis of lily (Morohashi et al., 2003); and the DLT subfamily in BR signaling in rice (Tong et al., 2009). AtSHR and AtSCR subfamily members participate in root radial patterning and growth in *Arabidopsis* by forming a SCR/SHR complex, while AtSCL3 subfamily members integrate multiple signals during root cell elongation (Di Laurenzio et al., 1996; Helariutta et al., 2000; Heo et al., 2011).

A large number of *GRAS* genes are associated with responses to abiotic stresses. For example, *SCL14* activates a general detoxification network in *Arabidopsis* in response to xenobiotics (Fode et al., 2008). *GRAS1* participates in signal transduction pathways in tobacco and increases the level of reactive oxygen species under various stress conditions (Mayrose et al., 2006; Fode et al., 2008). Overexpression of *PAT1*, a GRAS member from wild grape, enhances abiotic stress tolerance in *Arabidopsis* (Czikkel and Maxwell, 2007). The SCL4/7 subfamily members in rapeseed appear to be involved in enhancing drought and salt tolerance (Yuan et al., 2016). *GRAS6*-silenced tomato plants have reduced tolerance to drought stress (Mayrose et al., 2006). *GRAS23* is involved in drought resistance and oxidative stress tolerance as well as decreased hydrogen peroxide (H_2_O_2_) accumulation *via* regulation of stress-related gene expression in rice (Xu et al., 2015). In tomato, the *GRAS40* gene is essential for the activation of abiotic stress-inducible promoters and auxin and gibberellin signaling (Liu et al., 2017).

Although previous research has revealed the role of *GRAS* genes in responses to abiotic stresses, up to now, there have been few reports of *GRAS* genes involved in abiotic stress in soybean (*Glycine max*). In this study, we performed a comprehensive genome-wide analysis of the *GRAS* gene family in soybean and surveyed the characteristics of 117 *GRAS* genes. *GmGRAS37*, which was significantly upregulated under drought and salt stress conditions and abscisic acid (ABA) and brassinosteroid (BR) treatments, was chosen for further analysis. Overexpression of *GmGRAS37* improved soybean tolerance to drought and salt stress, indicating the importance of the *GmGRAS37* gene in abiotic stress responses.

## Materials and Methods

### Identification of *GRAS* Genes in Soybean

GmGRAS protein sequences were obtained from Phytozome (Zhang et al., 2012). The Hidden Markov Model (HMM) profile corresponding to the GRAS domain (PF03514; Lu et al., 2015) from the Pfam protein family database was used to scan the predicted proteins in the soybean genome (*G. max* Wm82.a2.v1) using HMMERv3 (Prince and Pickett, 2002). The soybean GRAS protein sequences were aligned using the HMM model in HMMERv3. The putative GRAS gene core sequences were verified by performing searches against the Pfam and SMART databases to confirm the presence of the GRAS conserved domain. The *Arabidopsis GRAS* gene family protein sequences and annotation information were downloaded from TAIR (Liu and Widmer, 2014), and the protein sequences of maize and rice were obtained from previous studies (Liu and Widmer, 2014; Guo et al., 2017). Molecular weight and isoelectric point information for GmGRASs were obtained from the ExPASy online website (Supplementary Table S1).

### Chromosomal Location and Phylogenetic Analysis

The physical locations of *GRAS* genes on soybean chromosomes were extracted from the soybean genomic database in Phytozome. All *GmGRASs* were mapped onto the 20 chromosomes of soybean.

The amino acid sequences of *Arabidopsis*, maize, rice, and soybean GRASs were aligned using ClustalW. A phylogenetic tree was constructed in MEGA version 7.0 (Kumar et al., 2016) using the maximum likelihood (ML) method with 1,000 bootstrap replications.

The amino acid sequence of nine GRAS proteins from tomato, rice, *Brassica rapa*, and soybean aligned using DNAMAN.

### Analysis of Gene Structure and the GRAS Motif

The intron insertion sites in the *GRAS* genes were identified by comparing the coding sequence with the corresponding full-length sequence using the Gene Structure Display Server (Guo et al., 2007). The conserved GRAS motifs were analyzed using the MEME online program; the maximum number of motifs was set to 15 (Bailey et al., 2009).

### Gene Duplication

BLASTp (*E*-value > 1e^−10^) was used to search all GRAS proteins of soybean. Duplicated genes were identified as described in a previous study; for each gene pair, when the alignment covered >80% of the longer gene and the aligned region had >80% identity at the nucleotide level, the pair was defined as duplicated genes (Fan et al., 2019). Tandem duplication events were determined by comparing the chromosomal position each duplicated gene. TBtools software was used to obtain and visualize the related syntenic blocks and duplicate gene pairs in soybean (Supplementary Table S2).

### Expression Patterns of *GRAS* Genes

The RNA-seq data for soybean *GRAS* genes in distinct tissues at different developmental stages under normal conditions were extracted from SoyBase (Du et al., 2018). The transcriptome data for various abiotic stresses were obtained in our previous study (Shi et al., 2018). TBtools software was used to visualize the expression levels of *GmGRASs*. The RNA-seq data are provided in Supplementary Tables S3–S7.

### Promoter Sequence Analysis

The promoter sequences (the 1,500 bp region upstream of the ATG start codon) of the *GmGRAS* genes were obtained from the Phytozome database and analyzed using the PlantCARE database (Lescot et al., 2002).

### Plant Materials and Stress Treatments

Soybean variety, Williams 82, was used for gene expression pattern analysis. The seeds were cultivated for 15 days in pots containing mixed soil (1:1 vermiculite/humus). For drought treatment, the soybean seedlings were placed on filter paper for 0, 0.5, 1, 2, 4, 8, 12, and 24 h. For the NaCl, ABA, and BR treatments, the soybean seedlings were immersed in 250 mM NaCl, 100 μM ABA, and 150 μM BR solution, respectively, for 0, 0.5, 1, 2, 4, 8, 12, and 24 h. After treatment, leaves were submerged immediately in liquid nitrogen and then stored at −80°C for further analysis (Zhang et al., 2019).

### RNA Extraction and qRT-PCR

Total RNA was isolated from soybean leaves with Trizol according to the manufacturer’s protocol (TIANGEN, Beijing, China). The cDNA was synthesized using the PrimeScriptTM RT Reagent Kit (TaKaRa, Shiga, Japan) according to the manufacturer’s protocol (Gao et al., 2019). The primers were designed using Primer Premier 5.0. The soybean Actin (*U60506*) gene was used as the internal control. Three biological replicates were used for quantitative real-time PCR (qRT-PCR) analysis (Le et al., 2011). All primers are listed in Supplementary Table S8.

### Subcellular Localization Analysis

The full-length cDNA sequence of *GmGRAS37* was fused to the N-terminus of the *hGFP* gene with expression driven by the CaMV 35S promoter. The 35S::GmGRAS37-hGFP fusion construct was transformed into *Arabidopsis* protoplasts by PEG4000-mediated transformation (Yoo et al., 2007). GFP expression in different subcellular compartments was detected by laser scanning confocal microscopy after 16 h at 22°C in darkness, as described elsewhere (Liu et al., 2013). Three biological replicates were performed in this experiment.

### *Agrobacterium Rhizogenes*-Mediated Transformation of Soybean Hairy Roots

Soybean Williams 82 was used for *Agrobacterium rhizogenes*-mediated transformation to generate *GmGRAS37*-overexpressing (*GmGRAS37*-OE) soybean hairy roots. The cDNA of *GmGRAS37* was ligated into the plant transformation vector pCAMBIA3301 under the control of the CaMV *35S* promoter. For the *GmGRAS37* RNA interference (*GmGRAS37*-RNAi) construct, a 586 bp fragment consisting of a 220 bp *GmGRAS37* fragment and its antisense sequence and a 146 bp maize alcohol dehydrogenase gene as connection between the repeats was synthesized (Augct, China) and inserted into pCAMBIA3301. The recombinant constructs and empty pCAMBIA3301 vector (CK) were transferred into *A. rhizogenes* strain K599 and then injected into hypocotyls following the protocol described previously (Kereszt et al., 2007; Du et al., 2018). The injected plants were placed in a greenhouse and kept at high humidity until hairy roots were generated at the infection site and had grown to about 5 cm in length. Remove the hypocotyl below 1 cm of the infected site. Seedlings were transplanted into mixed soil (1:1 vermiculite/humus) and cultured normally in the greenhouse for 7 days (25°C 16 h light/8 h dark photoperiod). After verification, positive soybean hair roots were used for abiotic stress assays, with six biological replicates of each stress treatment. For drought treatment, the soybean plants were grown without watering for 7 days, and for the NaCl treatment, the soybean plants were treated with 250 mM NaCl for 3 days. The primers used for cloning are listed in Supplementary Table S8.

### Measurement of Physiological Indicators

The leaves of drought‐ and salt-treated *GmGRAS37*-RNAi, EV-Control, and *GmGRAS37*-OE seedlings were obtained for measuring physiological indicators. The catalase (CAT), peroxidase (POD), and superoxide dismutase (SOD) activities and the malonaldehyde (MDA) content of leaves were determined using the corresponding assay kits (Cominbio, Suzhou, China) based on the manufacturer’s protocols. The measurement of chlorophyll content and determination of relative electrical conductivity (REC) were carried out as described previously (Sharp et al., 1990). All measurements were from three biological replicates.

### Trypan Blue and Nitroblue Tetrazolium Staining

The leaves of *GmGRAS37*-RNAi, EV-Control, and *GmGRAS37*-OE seedlings subjected to drought or NaCl treatment were soaked in 0.5% trypan blue (BioDee, China) and nitroblue tetrazolium (NBT; Creek Huizhi, China) solution for 12 h and then in 75% ethanol for decoloration until the samples became white (Du et al., 2018). Images were taken with a Canon 50D (Canon, Japan) camera. Each experiment was performed in triplicate.

## Results

### Identification of *GRAS* Genes in the Soybean Genome

A total of 118 *GRAS* genes were discovered in the soybean genome. All of the encoded GRAS proteins were checked for the presence of the GRAS domain using the SMART and Pfam databases; 117 genes contained a GRAS domain. The 117 *GRAS* genes were named *GmGRAS1* to *GmGRAS117* according to their chromosomal positions (Supplementary Table S1). The protein lengths, molecular weights, and isoelectric points are provided in Supplementary Table S1. Of the 117 GmGRAS proteins, the protein length of GmGRAS from 168 (GmGRAS56) amino acids to 842 amino acids (GmGRAS112). The smallest protein Mw is 18975.84 Da (GmGRAS56), and the largest protein Mw is 91543.91 Da (GmGRAS112). The pI from 4.76 (GmGRAS34) to 9.21 (GmGRAS56).

### Chromosomal Distribution, Phylogenetic Analysis, and Multiple Sequence Alignment

**Figure 1:**
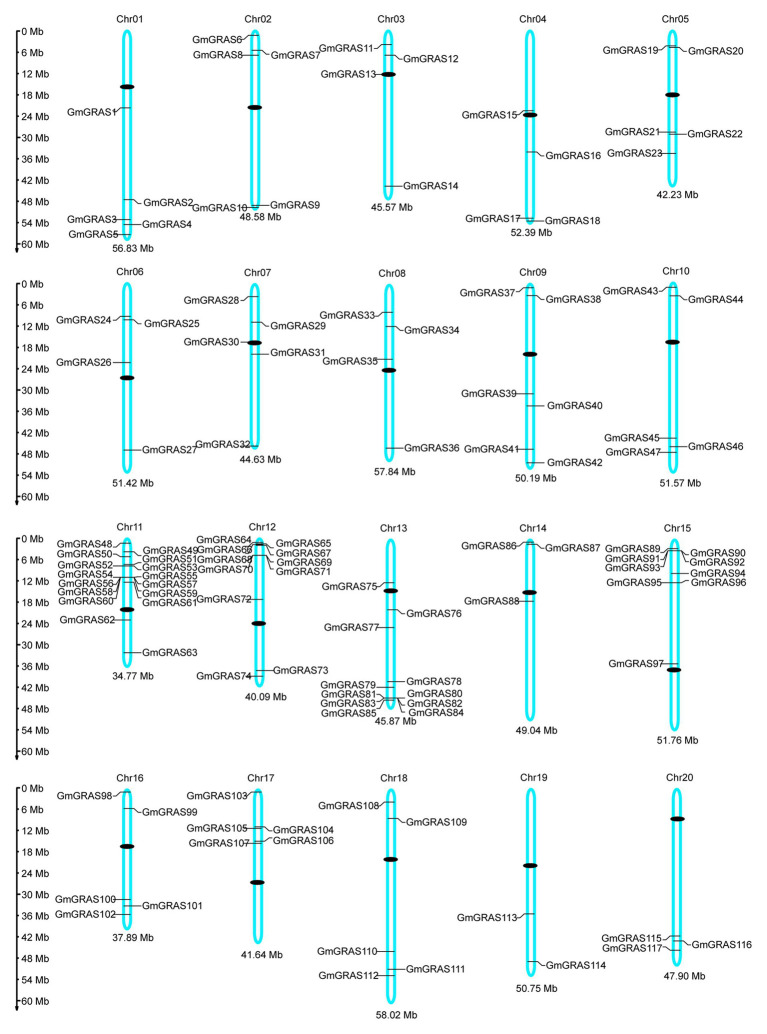
Chromosomal distribution of 117 *GRAS* genes in soybean. The scale bar on the left indicates the size of the chromosomes.

The physical location map of the *GmGRASs* was drawn based on the physical location information of the soybean genome. The 117 *GRAS* genes were widely and irregularly distributed on the 20 soybean chromosomes (Figure 1). Chromosome 11 harbored the most *GmGRAS* genes (16 genes), followed Chromosome 12 and Chromosome 13 (11 genes each). Chromosome 19 contained the fewest *GRAS* genes (two genes). In addition, we found that the number of *GRASs* distributed in the middle of the 20 chromosomes in soybean was relatively small, and its distribution on chromosomes was similar to *AtGRASs* and *OsGRASs* (Tian et al., 2004).

**Figure 2:**
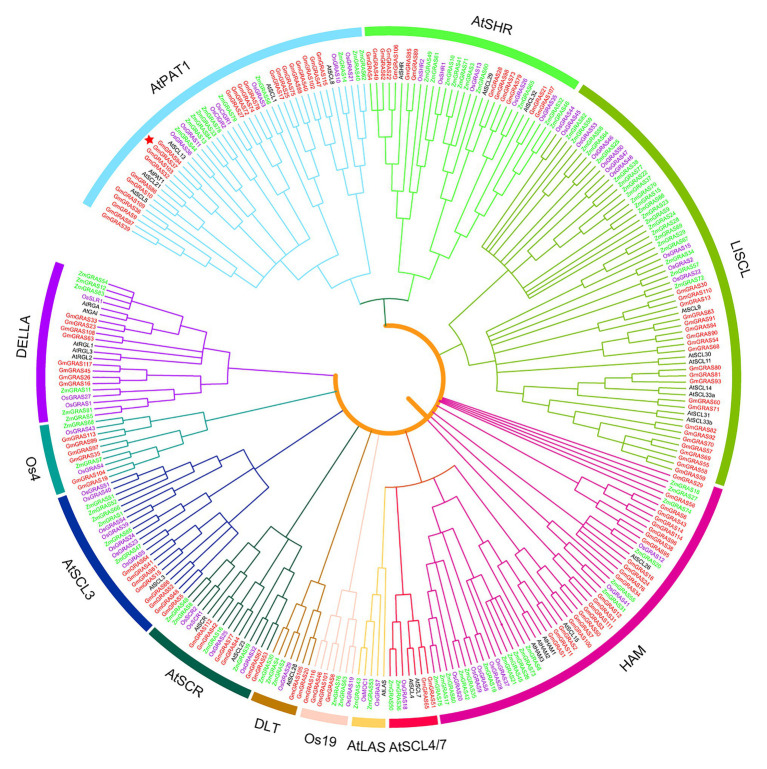
Phylogenetic tree of GRAS proteins from soybean, Arabidopsis, maize, and rice. The complete amino acid sequences of GRAS proteins were aligned by ClustalW, and the phylogenetic tree was constructed using the maximum-likelihood method in MEGA7. The ten groups are represented by different colors.

To explore the phylogenetic relationships of *GRAS* genes in different plant species, we built phylogenetic trees from the alignment of 286 GRAS domain amino acid sequences from soybean (117), *Arabidopsis* (33), maize (86), and rice (50) using the ML method in MEGA7.0 (Figure 2). In the resulting tree, the *GRAS* genes were divided into 12 subfamilies, in which 11 contained soybean *GRAS* genes: DELLA, DLT, HAM, AtPAT1, LISCL, AtSCR, AtSCL3, AtSHR, AtSCL4/7, Os19, Os4, and AtLAS. These subfamilies were designated following previous studies (Liu and Widmer, 2014; Guo et al., 2017). The HAM and LISCL groups were the two largest subfamilies. In general, members of most of the GRAS subfamilies were found in all four species. However, the soybean GRAS family does not include the AtLAS subfamily, and Os4 and Os19 subfamilies did not contain any *Arabidopsis* genes, which indicating that lineage-specific gene loss had occurred in soybean and *Arabidopsis*. The lineage-specific genes may represent genes with major diversity in the function of a particular species, genes that may have been highly specialized, or genes that have been lost from other species.

**Figure 3:**
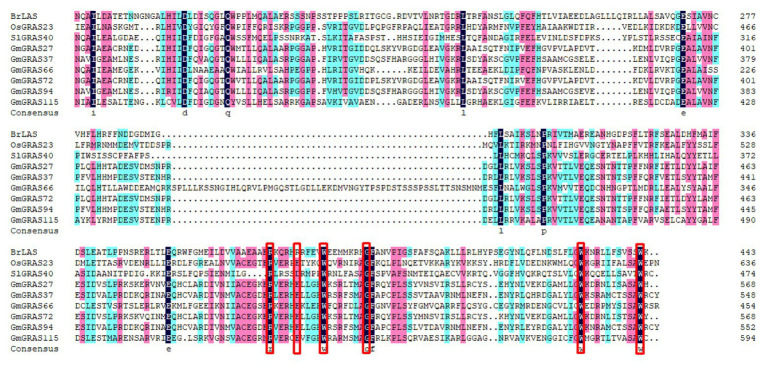
Multiple sequence alignment of nine GRAS proteins from tomato, rice, *Brassica rapa*, and soybean. Amino acid sequences were aligned using DNAMAN. Black shading represents 100% amino acid similarity, blue shading represents >75% similarity, and pink shading represents >50% similarity. The red rectangles indicate residues R-E, W-G, and W-W.

To explore the conservation between the GmGRAS proteins and BrLAS, OsGRAS23, and SlGRAS40, which have been reported to be involved in stress tolerance (Xu et al., 2015; Liu et al., 2017; Li et al., 2018), and GmGRAS27, GmGRAS37, GmGRAS66, GmGRAS72, GmGRAS94, and GmGRAS115, which are expressed at high levels in the two transcriptome databases of soybean drought and salt, were selected for multiple sequence alignment. The alignment of the C-terminal regions of these nine proteins indicated that the GRAS proteins were relatively highly conserved (Figure 3). All nine proteins contained the SAW motif (Figure 3), which is characterized by three pairs of absolutely conserved residues: R-E, W-G, and W-W (Pysh et al., 1999). Additionally, we constructed a phylogenetic tree to reveal the relationship between the nine proteins and GmGRASs are more closely related to each other. The closer the relationship between proteins, the higher the protein sequence similarity (Supplementary Figure S1).

### Gene Structure and Motif Composition of Soybean *GRAS* Genes

**Figure 4:**
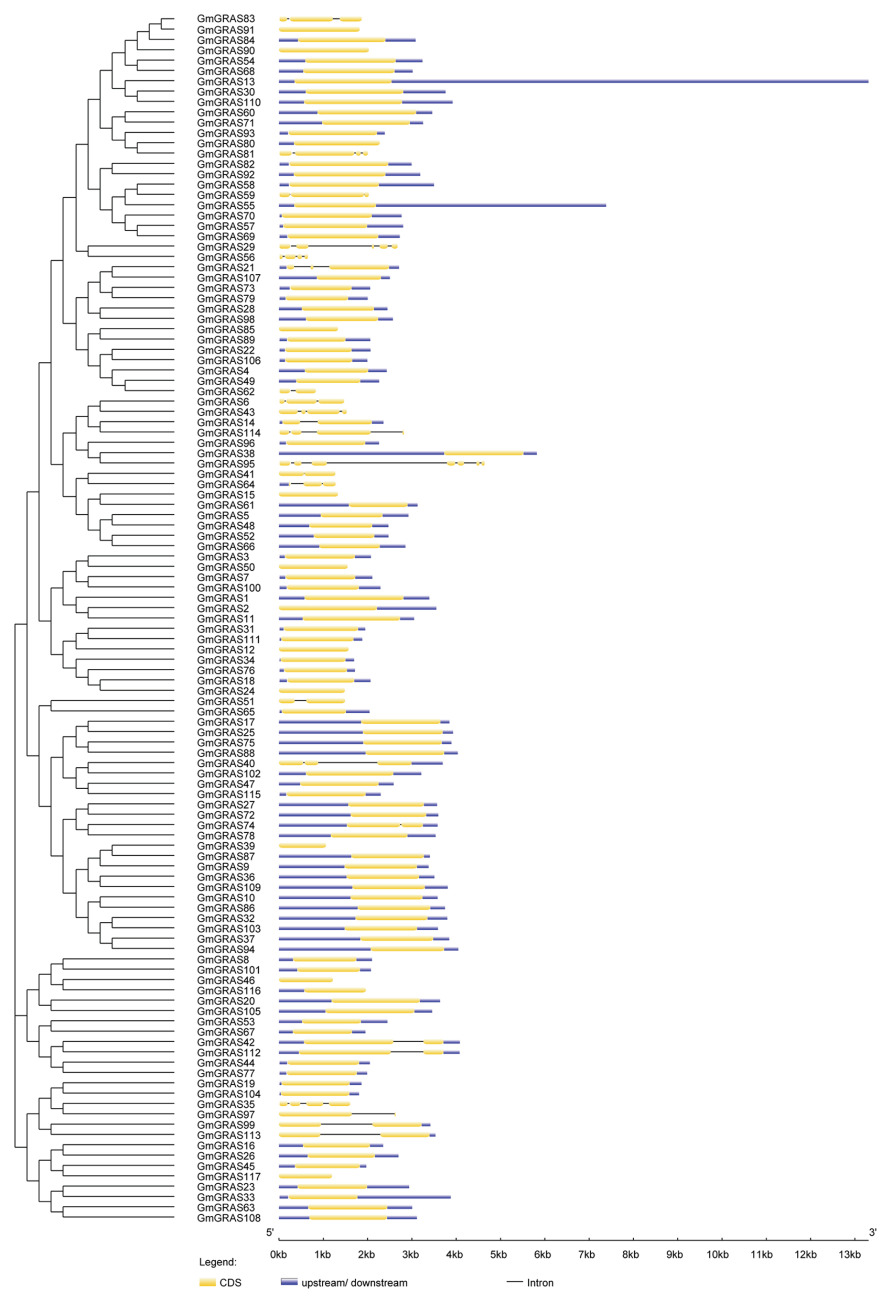
Phylogenetic relationships and structures of the 117 GmGRAS proteins. The phylogenetic tree was constructed using MEGA7.0 software; the different classes of GRAS proteins make up separate clades. The schematic diagram indicates the gene structure. Introns and exons are indicated by black lines and yellow boxes, respectively. The lengths of introns and exons of each gene are displayed proportionally.

To identify differences in gene structure, the exon and intron structures of the 117 soybean *GRAS* genes were compared; this analysis provided useful evidence for the evolution of structural diversity in the GRAS family. Almost all *GRAS* genes contained very few or no introns (Figure 4 and Supplementary Table S1); 80.34% of the *GRAS* genes was free of introns, which is similar to the lack of introns in members of this family in other species. For example, 88, 83.3, and 80.23% of *GRAS* genes in grapevine, Chinese cabbage, and maize, respectively, have no introns (Song et al., 2014; Guo et al., 2017). Four and six introns were found in *GmGRAS29* and *GmGRAS95*, respectively.

**Figure 5:**
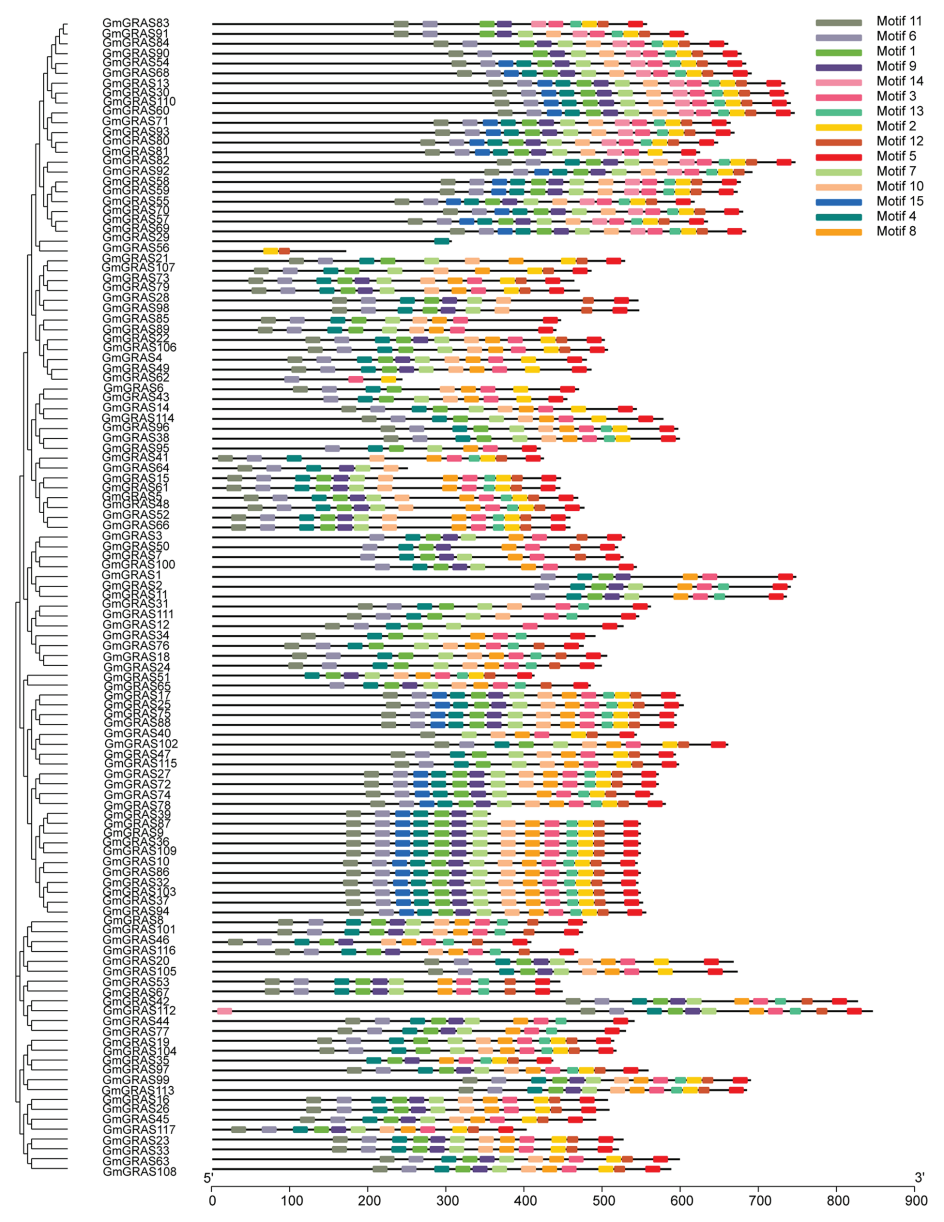
Putative motifs in each GmGRAS protein. Conserved motifs were identified using MEME and TBtools software. Ten putative motifs are indicated by colored boxes. The length of each protein can be estimated using the scale at the bottom.

Conservation of motifs in the 117 *GmGRAS* proteins was analyzed using the MEME website (Figure 5). A total of 15 different conserved motifs were discovered. Because the structures and functions of the GRASs are not known, the motifs were defined based on sequence conservation. The C-terminal regions contained a highly conserved domain (motif 5), and the sequence ZGCLLLGWKGRPLIAASAWR was found in most GRAS proteins (Supplementary Figure S2). Six proteins did not contain this conserved motif, namely *GmGRAS92*, *GmGRAS29*, *GmGRAS56*, *GmGRAS62*, *GmGRAS64*, and *GmGRAS39*, which may be because the C-terminal regions of these GRAS proteins are truncated and missing a part of the GRAS domain.

### Duplication and Divergence Rate of Soybean *GRAS* Genes

**Figure 6:**
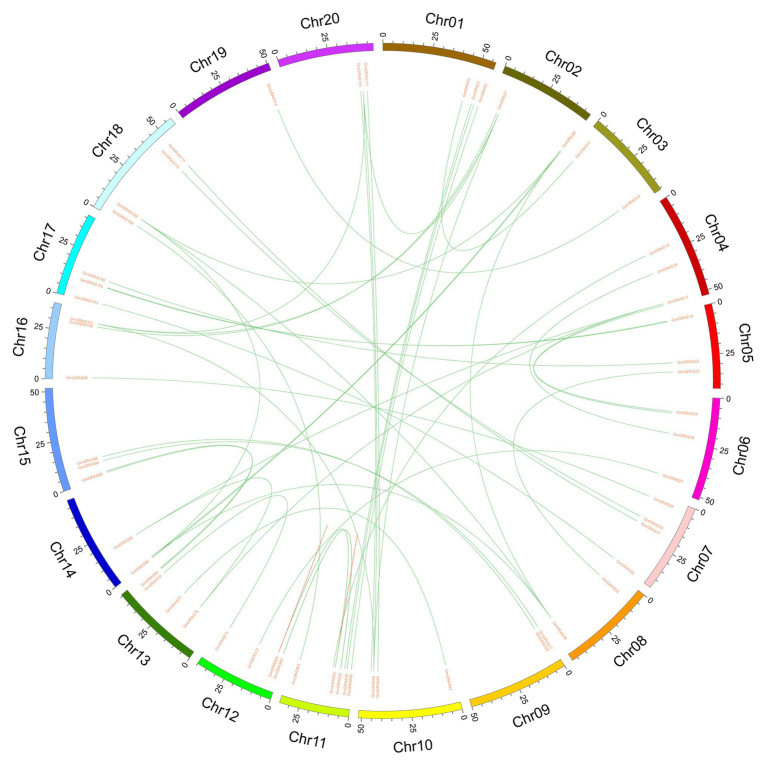
Distribution of segmentally duplicated *GmGRAS* genes on soybean chromosomes. Green lines indicate duplicated *GRAS* gene pairs.

We analyzed the duplication events giving rise to *GmGRAS* genes because gene duplication plays an important role in the amplification of gene families and their subsequent evolution. When two or more genes were located within a 200 kb chromosomal region, they were deemed to have arisen from tandem duplication events (Holub, 2001). Among the *GmGRASs*, six genes were clustered into seven tandem duplicated regions on soybean chromosomes 11 and 12, and 54 pairs of segmentally duplicated genes were detected; these segmental duplicates were found on all 20 chromosomes. (Figure 6 and Supplementary Table S2). The results showed that gene segmental duplication events may have been the main driving force behind *GRAS* gene evolution in soybean.

**Figure 7:**
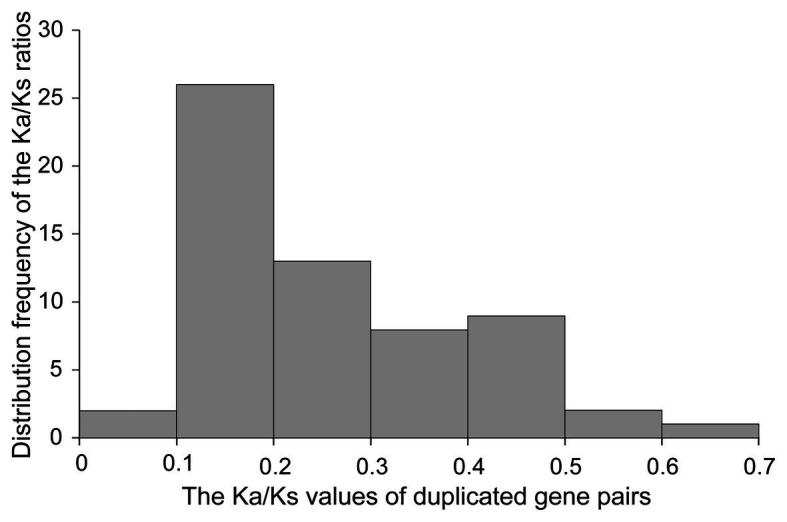
Histogram of distribution frequency of pairwise Ka/Ks ratios for pairs of homologous genes.

We estimated the synonymous (Ks) and nonsynonymous (Ka) substitution rates (Ka/Ks) of 61 segmentally duplicated pairs (Figure 7 and Supplementary Table S2). The Ka/Ks ratios for segmentally duplicated gene pairs ranged from 0.06 to 0.62 with an average of 0.26. Furthermore, the frequency distribution of the Ka/Ks ratios showed that more than 60% of duplicated gene pairs had ratios ranging from 0.1 and 0.3. The fact that these duplicated *GRAS* genes have Ka/Ks ratios lower than 1 indicates that they are under purifying selection.

### Expression Patterns of *GRAS* Genes

**Figure 8:**
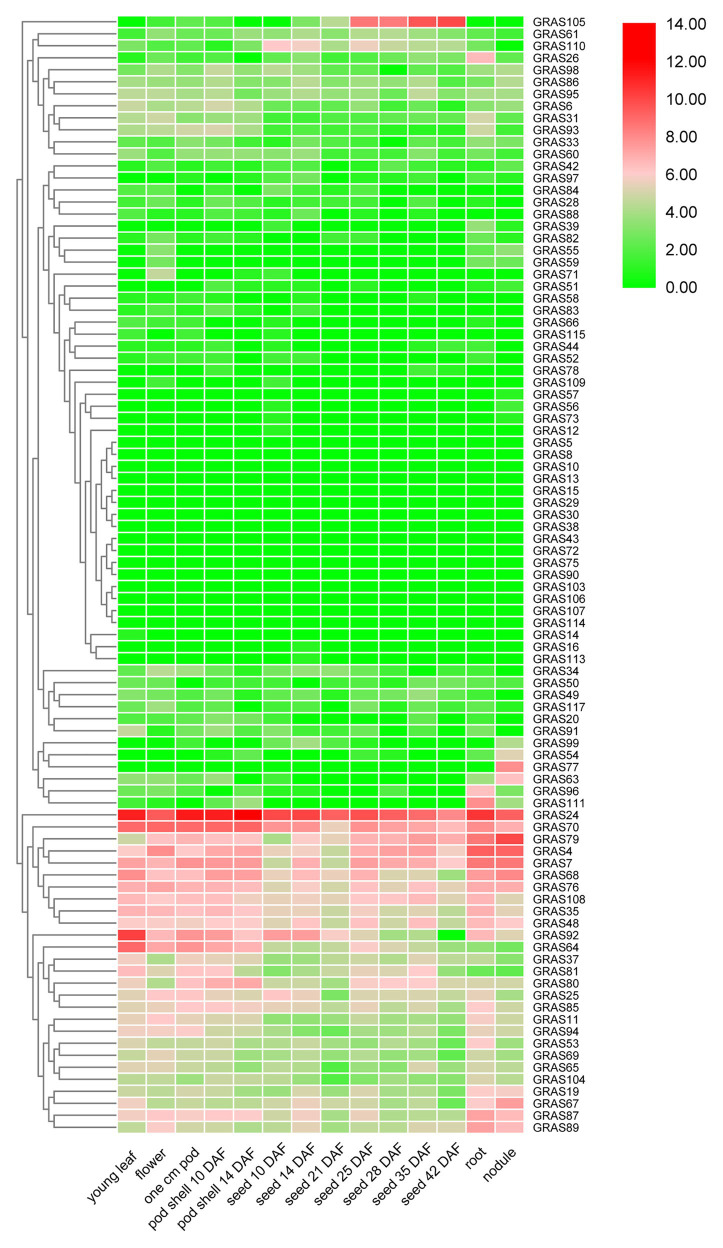
Heat map of the expression profiles of all *GmGRAS* genes in different soybean tissues. The expression abundance (in log10-based FPKM) of each transcript is represented by the color: red, higher expression; green, lower expression. Expression levels in 14 different tissues are shown: young leaves, flowers, one cm pods, pod shells at different days after flowering (DAF), roots, and nodules.

To investigate the expression profiles of the soybean *GRAS* members in different tissues, we used publicly available RNA-seq data from the SoyBase database, including young leaves, flowers, 1-cm pods, pod shells at different developmental stages, roots, and nodules. The expression levels of different *GRAS* genes varied widely in the same tissue (Figure 8 and Supplementary Table S3). Of the 117 *GRAS* genes, about one-fifth were not expressed. As shown in Figure 8, *GRAS24* and *GRAS70* showed extremely high expression levels in all tissues, suggesting that these *GRAS* genes are regulators of various processes of soybean growth and development. Also, we analyzed the expression levels of several duplicated genes of *GmGRASs* to understand the functional redundancy and homologous gene pairs (Figure 8). As a result, several duplicated genes pairs had similar expression levels (*GmGRAS37*/*94*, *GmGRAS85*/*89*, and *GmGRAS53*/*67*); however, *GmGRAS73*/*79*, *GmGRAS4*/*49*, and *GmGRAS92*/*82* displayed different or antipodal expression levels, which indicated that they may have experienced functional differences.

**Figure 9:**
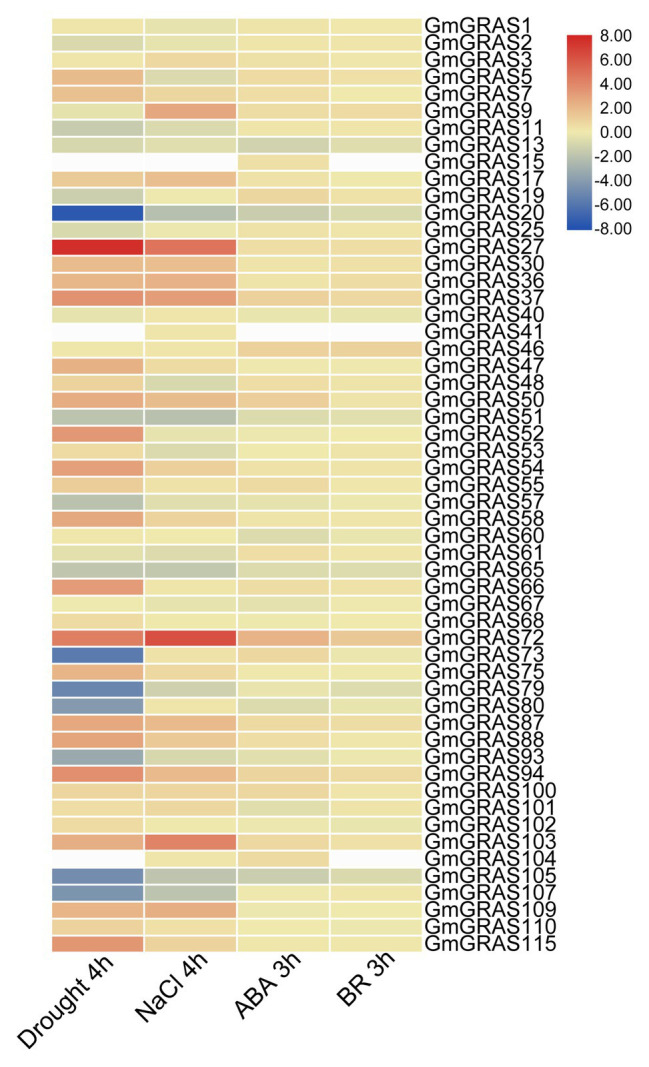
Heat map of expression profiles of all *GmGRAS* genes under different abiotic stresses. The expression abundance of each transcript (in log10-based FPKM) is represented by the color: red, higher expression; blue, lower expression; white, miss value.

Previous research has indicated that *GRAS* genes can be induced by various abiotic stresses (Mayrose et al., 2006; Xu et al., 2015; Liu et al., 2017). Analysis of RNA-seq data for soybean seedlings subjected to drought, NaCl, ABA, and BR treatments (Shi et al., 2018) revealed that 52, 54, 54, and 52 *GmGRAS* genes responded to drought, NaCl, ABA, and BR treatments, respectively (Figure 9 and Supplementary Tables S4–S7). Of these genes, 65.4% (34 out of 52), 64.8% (35 out of 54), 64.8% (35 out of 54), and 71.1% (37 out of 52) were upregulated under the drought, NaCl, ABA, and BR treatments, respectively. *GmGRAS27*, *GmGRAS37*, *GmGRAS66*, *GmGRAS72*, *GmGRAS94*, and *GmGRAS115* were all upregulated under all four conditions, so these six candidate genes were used for *cis*-elements and qRT-PCR analysis. Most duplicated gene pairs had similar expression levels, such as *GmGRAS27*/*72* and *GmGRAS37*/*94* under drought stress, *GmGRAS27*/*72* under salt stress, and *GmGRAS37*/*94* under exogenous ABA and BR treatments, which suggested that they might perform similar physiological functions.

### *Cis*-Elements Analysis

To investigate the biological functions of *GmGRAS* genes, six genes that have high transcription levels under drought, salt, ABA, and BR treatments according to the results of RNA-seq (*GmGRAS27*, *GmGRAS37*, *GmGRAS66*, *GmGRAS72*, *GmGRAS94*, and *GmGRAS115*) were selected for *cis*-elements analysis. The 1.5 kb region upstream of the start codon in promoter of each gene was isolated. A series of important *cis*-elements were identified (Table 1), including the ABRE (ABA-responsive element), MYB (drought responsive), MYC (drought and cold responsive), MBS (MYB-site), ERE (ethylene-responsive element), TCA-element (salicylic-responsive element), and GT-1 (salt induced). Moreover, these six genes had many MYB, MYC, and ABRE *cis*-elements, which suggested that these genes might respond to abiotic stresses, such as drought, ABA, and cold.

**Table 1:** Distribution and numbers of *cis*-acting elements in the promoters of soybean *GRAS* genes.

Genes	*GmRAS27*	*GmGRAS37*	*GmGRAS66*	*GmGRAS72*	*GmGRAS94*	*GmGRAS115*
ABRE	3	5	3	4	3	4
ARE	5	6	4	2	3	4
Box4	2	1	1	3	1	4
CGTCA-motif	5	4	0	3	0	4
ERE	1	1	4	1	0	2
G-Box	5	4	3	6	4	3
GT1-motif	3	1	2	3	0	4
LTR	2	1	0	1	1	4
MBS	2	2	1	1	2	0
MYB	8	3	8	16	7	4
MYC	5	6	7	9	6	1
P-box	0	2	4	1	2	0
TC-rich repeats	0	2	1	0	2	0
TCA-element	0	3	1	0	2	0
WUN-motif	0	0	2	0	0	1

### *GmGRAS* Genes Are Involved in the Response to Abiotic Stresses

**Figure 10:**
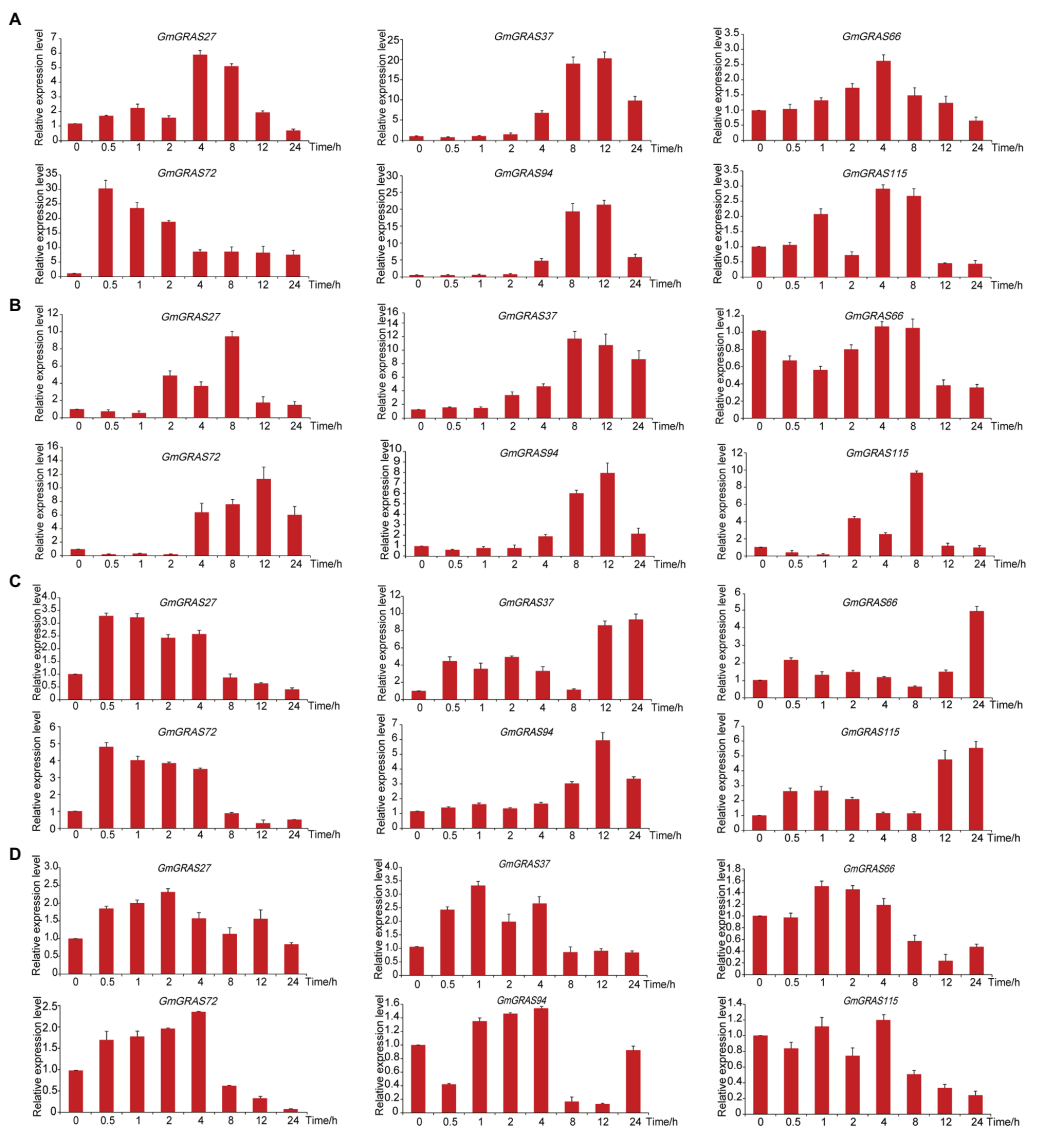
Expression patterns of *GmGRAS* genes under drought, salt, abscisic acid (ABA), and brassinosteroid (BR) treatment. **(A–D)** Expression levels of six *GmGRAS* genes, as measured using quantitative real-time PCR (qRT-PCR), under drought **(A)**, NaCl **(B)**, ABA **(C)**, and BR **(D)** treatment applied for 0, 0.5, 1, 2, 4, 8, 12, and 24 h. qRT-PCR data were normalized using the soybean Actin gene (U60506) and are displayed relative to 0 h. The *x*-axes show the duration of treatment and *y*-axes depict relative expression level (error bars indicate SD). The data are shown as means of three biology repeats ± SD.

The transcription levels of the six *GmGRAS*s under abiotic stress were further confirmed by qRT-PCR (Figure 10). The expression patterns determined by qRT-PCR corresponded to those from RNA-seq data. All six genes showed the same expression patterns under drought treatment and were upregulated by salt stress. In addition, the expression levels of these six genes were induced by ABA and BR. For example, *GmGRAS37*, which was highly expressed in the drought, NaCl, ABA, and BR RNA-seq datasets, displayed high expression under these conditions in qRT-PCR analysis. In addition the expression level of *GmGRAS37* was highest under salt (8 h), ABA (2 h), and BR (1 h) treatment than other genes, so *GmGRAS37* were selected for the further analysis. The expression patterns of these stress-induced *GmGRASs* provide useful information for further understanding their functions in coping with abiotic stresses.

### GmGRAS37 Is Localized in the Plasma Membrane, Nucleus, and Cytosol

**Figure 11:**
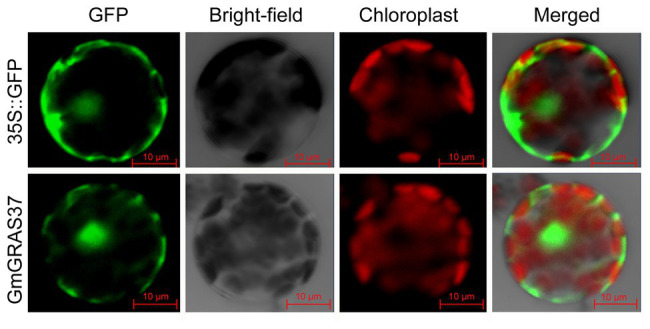
Subcellular localization of GmGRAS37-16318hGFP fusion protein. 35S::GFP was used as a control. The scale bar indicates 10 μm.

*GmGRAS37* was selected for further analysis because it was significantly upregulated under all tested treatment conditions. To analyze the subcellular localization of GmGRAS37, the cDNA of *GmGRAS37* lacking the stop codon was fused to the N-terminus of the *hGFP* reporter gene and ligated into an expression vector under the control of the CaMV 35S promoter. The vector was transformed into *Arabidopsis* protoplasts and observed under a confocal microscope. The control hGFP and GmGRAS37-16318hGFP fusion proteins both localized to the plasma membrane, nucleus, and cytosol (Figure 11). These results indicated that GmGRAS37 may function throughout the cell.

### Overexpression of *GmGRAS37* Improves Drought and Salt Tolerance in Soybean Hairy Roots

**Figure 12:**
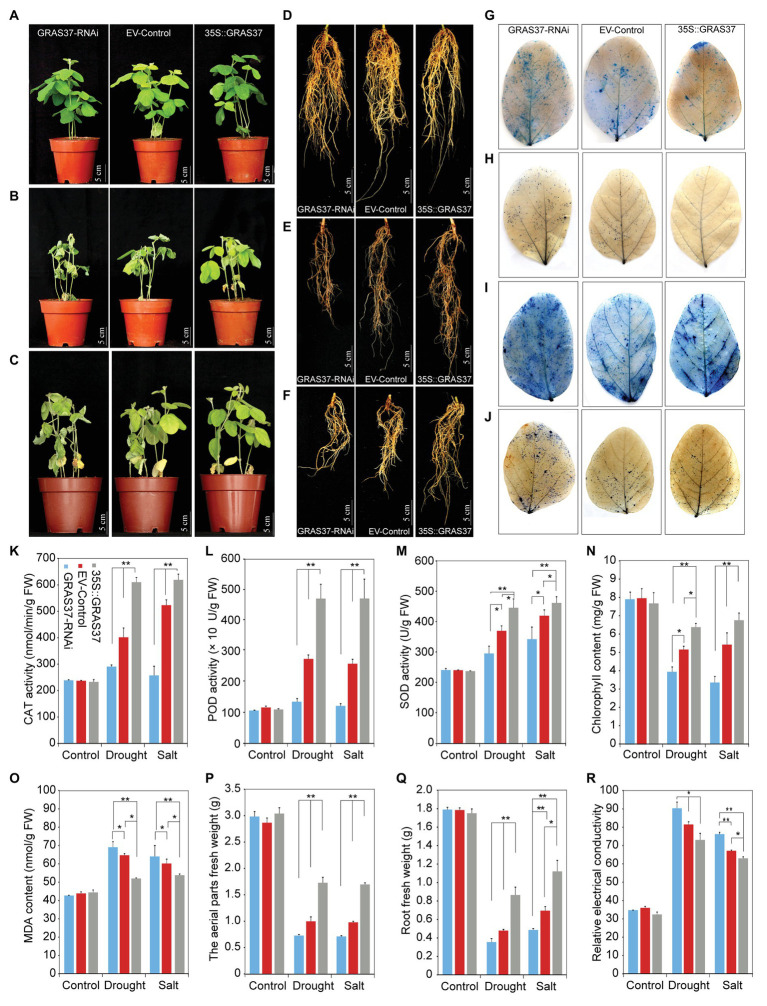
Analysis of the function of soybean *GmGRAS37* under normal conditions and drought and salt stresses. **(A–C)** Phenotypes of transgenic soybean hairy root composite *GRAS37*-RNAi, EV-Control (expressing the pCAMBIA3301 empty vector), and *GmGRAS37*-overexpression (*35S::GRAS37*) plants under normal conditions **(A)**, drought stress **(B)**, and salt stress **(C)**. **(D–F)** The roots of transgenic soybean hairy root composite *GRAS37*-RNAi, EV-Control, and *35S: GRAS37* plants under normal conditions **(A)**, drought stress **(B)**, and salt stress **(C)**. **(G,I)** Trypan blue staining of leaves of transgenic soybean hairy root composite *GRAS37*-RNAi, EV-Control, and *35S: GRAS37* plants under drought **(G)** and salt stress **(I)**; the dead cells can be strained, but living cells cannot. **(H,J)** Nitroblue tetrazolium (NBT) staining of the leaves of transgenic soybean hairy root composite *GRAS37*-RNAi, EV-Control, and *35S: GRAS37* plants under drought **(H)** and salt stress **(J)**. The intensity of color indicates the concentration of O_2_^−^ in the leaves. **(K–M)** CAT **(K)**, POD **(L)**, and SOD **(M)** activities of transgenic soybean hairy root composite *GRAS37*-RNAi, EV-Control, and *35S: GRAS37* plants under drought conditions. **(N,O)** Chlorophyll content **(N)** and MDA content **(O)** of transgenic soybean hairy root composite *GRAS37*-RNAi, EV-Control, and *35S: GRAS37* plants under drought conditions. **(P,Q)** The fresh weights of the aerial parts **(P)** and roots **(Q)** of transgenic soybean hairy root composite *GRAS37*-RNAi, EV-Control, and *35S: GRAS37* plants under drought conditions. **(R)** Relative electrical conductivity of transgenic soybean hairy root composite *GRAS37*-RNAi, EV-Control, and *35S: GRAS37* plants under drought conditions. Vertical bars indicate ±SD of three replicates. ∗ (*p* < 0.05) and ∗∗ (*p* < 0.01) indicate significant differences determined by Student’s *t*-test.

To further verify the physiological function of *GmGRAS37 in vivo*, *GmGRAS37*-OE, EV-transformed control, and *GmGRAS37*-RNAi hairy roots were generated using *A. rhizogenes*-mediated transformation (Gao et al., 2019). qRT-PCR analysis showed that the level of *GmGRAS37* expression in OE transgenic hairy roots was much higher than that in the EV-transformed control, and the level of *GmGRAS37* expression in RNAi transgenic hairy roots was lower than that in the EV-transformed control (Supplementary Figure S3). Under normal growth conditions, the *GmGRAS37*-OE transgenic plants displayed almost the same growth pattern as the *GmGRAS37*-RNAi and EV-Control plants (Figure 12A). However, under drought and salt stress conditions, there were obvious differences in the growth patterns of the *GmGRAS37*-RNAi, EV-Control, and *GmGRAS37*-OE plants (Figures 12B,C). Severely dehydrated leaves were observed on *GmGRAS37*-RNAi plants after drought and salt treatment, and wilted leaves appeared in EV-Control plants. However, the *GmGRAS37*-OE seedlings showed delayed and less leaf rolling during drought stress. There were no considerable differences between the *GmGRAS37*-RNAi, EV-Control, and *GmGRAS37*-OE roots under normal growth conditions (Figure 12D), but the *GmGRAS37*-OE roots were longer than EV-Control roots and the roots of *GmGRAS37*-RNAi were shorter than those of the EV-Control under drought and salt treatment conditions (Figures 12E,F).

Trypan blue was used to observe cell activity in *GmGRAS37*-RNAi, EV-Control, and *GmGRAS37*-OE leaves. Under normal growth conditions, there was no significant difference in trypan blue staining (Supplementary Figure S4A). Under drought and salt treatment conditions, less staining was observed in *GmGRAS37*-OE than in the EV-Control. In contrast, more intense staining was observed in *GmGRAS37*-RNAi leaves compared with EV-Control leaves (Figures 12G,I). These results suggested that the cell membrane integrity and stability in the *GmGRAS37*-OE plants leaves were better than those in the EV-Control and *GmGRAS37*-RNAi leaves. NBT was used to assess the level of superoxide anions (O_2_^−^), which affect plant growth and development. No difference was observed between the *GmGRAS37*-RNAi, EV-Control, and *GmGRAS37*-OE leaves under normal growth conditions (Supplementary Figure S4B). Under drought and salt treatment conditions, the amount of NBT staining was lower in *GmGRAS37*-OE leaves than in EV-Control leaves. However, significantly darker staining was observed in *GmGRAS37*-RNAi leaves than in EV-Control leaves (Figures 12H,J). These results indicated that the content of O_2_^−^ in *GmGRAS37*-OE was lower than that in EV-Control plants; however, the content of O_2_^−^ in *GmGRAS37*-RNAi was greater than that in EV-Control plants.

We found that the CAT, POD, and SOD activities and chlorophyll content of *GmGRAS37*-OE plants under drought and salt treatment conditions were all greater than those in EV-Control plants but lower in *GmGRAS37*-RNAi plants than in EV-Control plants (Figures 12K–N). In contrast, the MDA content in *GmGRAS37*-OE plants was lower than that in EV-Control plants, and the content in *GmGRAS37*-RNAi plants was greater than that in EV-Control plants (Figure 12O). Compared with EV-Control plants, the aerial tissue and root biomasses of *GmGRAS37*-OE plants were higher under drought and salt stresses, while those of *GmGRAS37*-RNAi plants were lower (Figures 12P,Q). Under drought and salt stresses, *GmGRAS37*-RNAi had a higher REC than EV-Control plants, and *GmGRAS37*-OE the lowest value (Figure 12R).

### Analysis of the Mechanism of *GmGRAS37*-Mediated Resistance in Soybean

**Figure 13:**
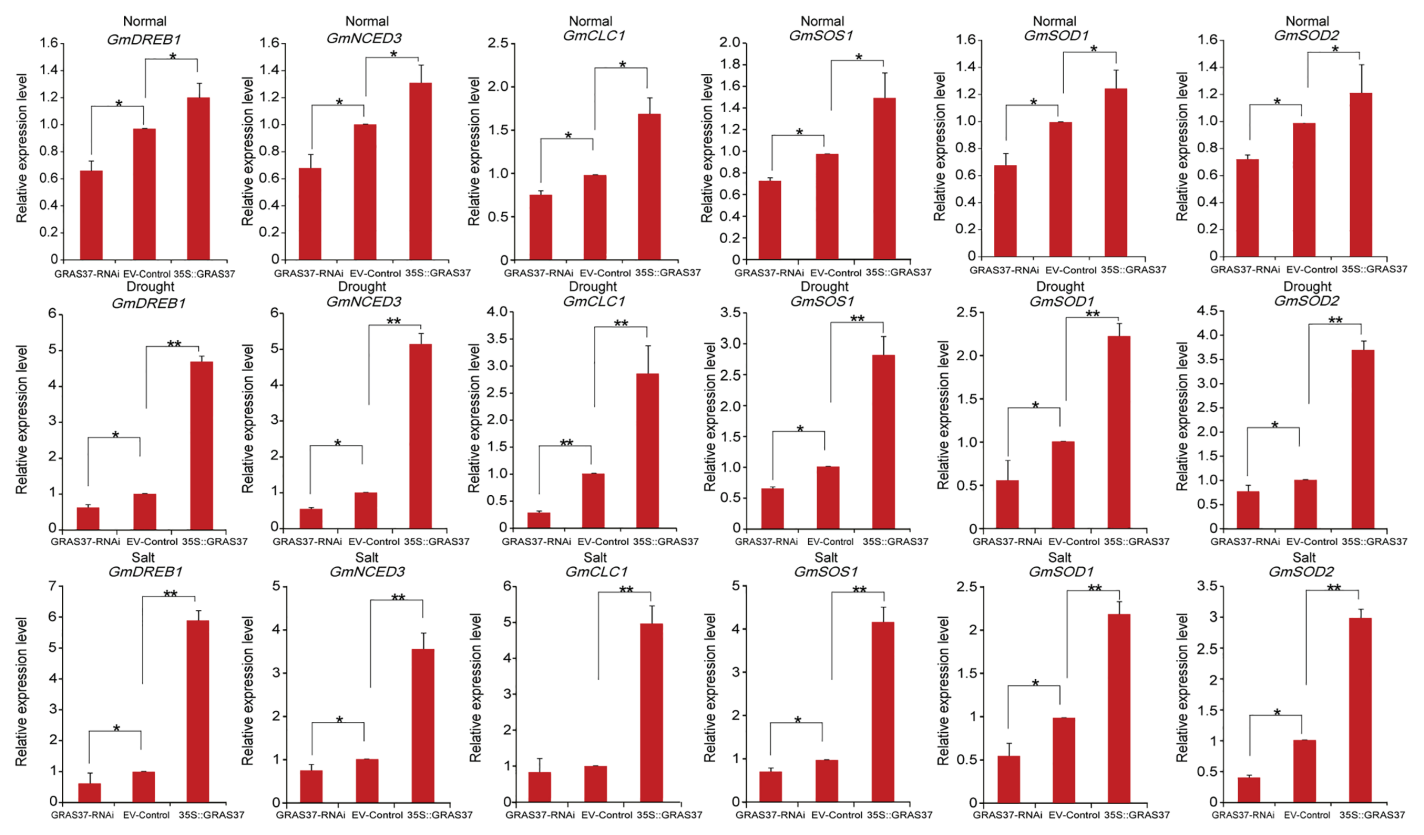
Expression levels of six stress-responsive genes in transgenic *GmGRAS37* soybean hairy root plants under normal conditions, drought stress, and 250 mM NaCl treatment determined by qRT-PCR. Vertical bars indicate ±SD of three replicates. ∗ (*p* < 0.05) and ∗∗ (*p* < 0.01) indicate significant differences determined by Student’s *t*-test.

To analyze the possible resistance mechanisms regulated by *GmGRAS37* during multiple stress responses, *GmGRAS37*-OE, EV-Control, and *GmGRAS37*-RNAi hairy roots treated with or without stresses were used for investigating the expression changes of six genes (*GmDREB1*, *GmNCED3*, *GmCLC1*, *GmSOS1*, *GmSOD1*, and *GmSOD2*) that were reported to be involved in abiotic stress responses (Kidokoro et al., 2015; Wei et al., 2016; Cao et al., 2018; Li et al., 2019; Yadav et al., 2019) and identified as being upregulated in response to stress in our analysis of *de novo* transcriptomic sequences of soybean. The results of qRT-PCR analysis (Figure 13) showed that under normal growth conditions, the transcript levels of these genes in *GmGRAS37*-OE plants were higher than those in EV-Control plants; however, the transcript levels were lower in *GmGRAS37*-RNAi plants than in EV-Control plants. Similarly, the transcript levels of these six genes in *GmGRAS37*-OE plants were dramatically upregulated under drought and salt stresses compared with the EV-Control plants; however, the transcript levels of these genes in *GmGRAS37*-RNAi plants were lower than those in EV-Control plants. These results showed that *GmGRAS37* may activate the transcription of drought‐ or salt-responsive genes to meditate stress responses.

## Discussion

GRAS proteins, which are plant-specific transcription factors, play vital roles in various processes of tissue or organ development. The previous study about soybean GRAS family had been reported and identified 117 *GRAS* member genes (Wang et al., 2020). However, in our study, we found a new GRAS family member gene Glyma.07G105100 by scanning the whole soybean genome, and further analysis showed soybean Glyma.U013800 gene was not found on a total of 20 typical soybean chromosomes, so it was deleted. Therefore, 117 *GRAS* genes were identified in the soybean genome and used to system analysis in our study. One hundred and seventeen soybean genes were divided into nine subfamilies in the previous study (Wang et al., 2020); however, *GRAS* genes were classified into 12 subfamilies by introducing maize and rice *GRAS* genes, and among these 12 subfamilies, 11 contained soybean *GRAS* genes. By analyzing the intron-exon structure of *GmGRAS* genes, we found that the majority of these genes were free of introns, which was similar to the observed lack of introns in *Arabidopsis* and rice *GRAS* genes (Tian et al., 2004). A previous study showed that ancestors of each eukaryote had intron-rich genes and that extensive loss and insertion of introns from most genes may have occurred due to selective pressure, with gene duplication accelerating this process (Roy and Penny, 2007; Rogozin et al., 2012). Nevertheless, some *GRAS* genes have evolved different exon-intron structures, indicating that they likely evolved new specialized functions to adapt their environment. Previous research has indicated that the plant *GRAS* gene family originated from a prokaryotic genome through horizontal gene transfer, followed by duplication events (Zhang et al., 2012). Accordingly, the variation in the number of *GRAS* genes between species may be related to gene duplication events. In our study, we identified seven pairs of tandem duplicated *GmGRASs* and 54 pairs of segmentally duplicated *GmGRASs* (Figure 6 and Supplementary Table S2). These results further validate the notion that duplication plays a vital role in the expansion of the *GRAS* gene family and show that segmental duplication contributed more to the expansion of the soybean *GRAS* family than tandem duplication. Protein structure analysis showed that the GmGRAS27, GmGRAS37, GmGRAS72, GmGRAS94, and GmGRAS115 proteins all have the SAW motif, which is a highly conserved C-terminal region (Figure 3), and we found that these five GmGRAS proteins are in the same subfamily and have the same gene structure and motifs, which suggests that these *GmGRAS* genes may have similar functions.

*Cis*-acting elements analysis showed that there are many stress-related *cis*-acting elements in six *GmGRAS* genes with high transcription levels under drought, salt, ABA, and BR treatments (Table 1). Among these stress-related *cis*-acting elements, the ABRE is an important component in the ABA pathway and has been shown to be bound by transcription factors in response to ABA-mediated osmotic stress signals. Through qRT-PCR analysis (Figure 10), all six *GmGRAS* genes, which contain the ABRE, were upregulated by ABA. Further analysis found that the expression levels of these six *GmGRAS* genes were upregulated under drought and salt stress conditions. Therefore, it is speculated that these genes may be involved in abiotic stress response and participate in the regulation of ABA signaling. Our analysis showed that *GmGRAS37* was highly expressed under multiple stress conditions (Figure 10); therefore, this gene was selected for further analysis. Previous research has indicated that GRAS proteins, such as BrLAS, OsGRAS23, and SlGRAS40, play a significant role in growth and development and abiotic stress response in plants (Xu et al., 2015; Liu et al., 2017; Li et al., 2018). Protein structure analysis showed that the GmGRAS37 protein shared functional domains with these three stress response proteins. Further analysis revealed that drought and salt treatments resulted in significant differences in growth and physiology of *GmGRAS37*-RNAi, EV-Control, and *GmGRAS37*-OE plants. In particular, *GmGRAS37*-OE plants had significantly delayed leaf wilting; longer roots; higher CAT, POD, and SOD activities, chlorophyll content, and biomass; lower MDA content, REC, and H_2_O_2_ and O_2_^−^ levels; and fewer dead cells. The above results suggested that *GRAS37* may play a role in decreasing H_2_O_2_ accumulation *via* regulation of stress-related gene expression (Xu et al., 2015). Therefore, *GmGRAS37*-OE had increased stress resistance, whereas *GmGRAS37*-RNAi had impaired stress resistance.

Previous research indicated that several genes play an important role in drought and salt stresses. *GmDREB1*, DREB-type transcription factor, was reported to be strongly induced by multiple stresses, such as cold, drought, high salt, and heat. The GmDREB1 protein activates transcription by binding to dehydration-responsive elements (DREs). The *GmDREB1* gene can induce the expression of ABA receptor family genes. Previous studies have shown that *GmDREB1* can improve the drought tolerance of wheat (Kidokoro et al., 2015; Zhou et al., 2020). 9-*cis*-epoxycarotenoid dioxygenase (NCED) is considered to be an important contributor to ABA synthesis during drought and salt stress. Previous studies have indicated that seedlings overexpressing *OsNCED3* have increased drought stress tolerance (Hwang et al., 2010; Li et al., 2019). The chloride channel protein family mediates the transport of Cl^−^, which is important for plant nutrient supply, stomatal movement, hormone signal recognition and transduction, Cl^−^ homeostasis, and abiotic and biotic stress tolerance. *GmCLC1* enhances salt tolerance by reducing Cl^−^ accumulation to reduce the negative impact of salt stress (Wei et al., 2016). *GmSOS1* improves the salt tolerance of plants. Previous studies suggested that *GmSOS1* may play a role in the extrusion of Na^+^ from the roots and the regulation of long-distance Na^+^ transport from roots to shoots (Cao et al., 2018). *AtSODs* have been reported to improve tolerance to abiotic stresses such as salinity, cold, and drought stress; these stresses limit plant growth by causing an imbalance between the generation and metabolism of various reactive oxygen species. As a main component of the first line of defense, SOD converts O_2_^−^ into H_2_O_2_ and O_2_. SODs act as signals in various signal transduction pathways of plants and participate in various plant developmental processes (Yadav et al., 2019). Under drought and salt treatment conditions, these six genes were upregulated in *GmGRAS37*-OE plants, indicating that they are regulated by *GmGRAS37* in response to abiotic stress.

## Conclusion

A total of 117 soybean *GRAS* genes were identified and phylogenetically divided into 11 subfamilies. Six *GRAS* genes, including *GmGRAS37*, were induced by drought, salt, ABA, and BR treatments. *GmGRAS37* enhanced drought and salt tolerance in transgenic plants by activating the expression of *GmDREB1*, *GmNCED3*, *GmCLC1*, *GmSOS1*, *GmSOD1*, and *GmSOD2*. Our study provides a basis for further research on the functions of GRAS family members in abiotic stress tolerance.

## Data Availability Statement

The datasets presented in this study can be found in online repositories. The names of the repository/repositories and accession number(s) can be found in the article/Supplementary Material.

## Author Contributions

Z-SX coordinated the project, conceived and designed experiments, and edited the manuscript. T-TW performed experiments and wrote the first draft. T-FY revised the manuscript. H-GS, JC, Y-BZ, and MC contributed to data analysis and managed reagents. J-DF, W-LW, JG, and Y-ZM contributed with valuable discussions. All authors reviewed and approved the final manuscript.

### Conflict of Interest

The authors declare that the research was conducted in the absence of any commercial or financial relationships that could be construed as a potential conflict of interest.
