# Supplementary material for: Genome-Wide Analysis of the GRAS Gene Family and Functional Identification of GmGRAS37 in Drought and Salt Tolerance
Source: Front Plant Sci. 2020 Dec 23;11:604690. doi: 10.3389/fpls.2020.604690 (PMC7793673; doi:10.3389/fpls.2020.604690)
Supplement: Supplementary file 1 [file Data_Sheet_1.zip › Data Sheet 1.docx]

Supplementary Figure S1. A phylogenic tree of nine selected GRAS proteins.
