# Supplementary material for: Genome-Wide Analysis of the GRAS Gene Family and Functional Identification of GmGRAS37 in Drought and Salt Tolerance
Source: Front Plant Sci. 2020 Dec 23;11:604690. doi: 10.3389/fpls.2020.604690 (PMC7793673; doi:10.3389/fpls.2020.604690)
Supplement: Supplementary file 1 [file Data_Sheet_1.zip › Data Sheet 3.docx]

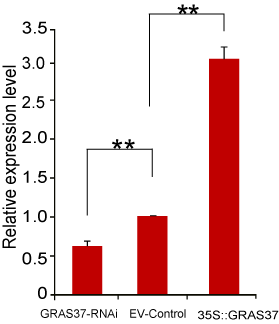


Supplementary **Figure S3.** qRT-PCR analysis of GmGRAS37 expression levels in GmGRAS37-OE, EV-Control, and GmGRAS37-RNAi transgenic hairy roots. The data are shown as means of three biology repeats ± SD.
